# Cortical thickness in youth with major depressive disorder

**DOI:** 10.1186/1471-244X-14-83

**Published:** 2014-03-20

**Authors:** Stephanie Reynolds, Normand Carrey, Natalia Jaworska, Lisa Marie Langevin, Xiao-Ru Yang, Frank P MacMaster

**Affiliations:** 1 Department of Psychiatry, University of Calgary, Behavioral Research Unit, Alberta Children’s Hospital, 2888 Shaganappi Trail NW, Calgary, AB T3B 6A8, Canada; 2 Department of Pediatrics, University of Calgary, Behavioral Research Unit, Alberta Children’s Hospital, 2888 Shaganappi Trail NW, Calgary, AB T3B 6A8, Canada; 3 Department of Psychiatry, Dalhousie University, Halifax NS, Canada

**Keywords:** Adolescence, Cortical thickness, Depression, Magnetic resonance imaging, Prefrontal cortex

## Abstract

**Background:**

Studies in adults with major depressive disorder (MDD) have implicated dysregulation of frontal-limbic circuits in the symptomology of this disorder. We hypothesized that the middle frontal gyrus (MFG; a core portion of the dorsolateral prefrontal cortex or DLPFC) and the anterior cingulate (caudal), regions implicated in emotive and cognitive control, would display a reduced cortical thickness in youth with MDD as compared to healthy, non-depressed adolescents.

**Methods:**

Sixteen healthy control adolescents (17.19 ± 1.87 years; 7 males, 9 females) and thirty MDD participants (16.89 ± 2.01 years; 9 males, 21 females) underwent magnetic resonance imaging (MRI). Cortical thickness analysis was carried out using FreeSurfer software.

**Results:**

Counter to our hypothesis, we observed thicker right and left rostral MFG in MDD adolescents as compared to controls (p = 0.004 and p = 0.005, respectively). Furthermore, the left caudal anterior cingulate cortex was thicker in MDD subjects as compared to controls (p = 0.009). In MDD subjects, there was a significant inverse correlation between age and left MFG thickness (r = -0.45, p = 0.001).

**Conclusions:**

These results have implications for the developmental trajectory of the frontal lobe in adolescent MDD. The MFG is implicated in the frontal-limbic circuits underlying executive functioning and their interaction with affective processing. Alterations in this region are likely involved with the symptoms of MDD. Limitations include a small sample size and cross sectional design.

## Background

Brain regions known to be associated with emotional regulation [1] are also implicated in the pathophysiology of major depressive disorder (MDD) in both adults and youth [2-8]. Neuroanatomical and functional disturbances within frontal and limbic regions, as well as their connective circuits, have been consistently noted in MDD, leading to the frontal limbic model of MDD [9]. Specifically, altered functioning of the anterior cingulate cortex (ACC), the dorsolateral prefrontal cortex (DLPFC) and the orbitofrontal cortex (OFC) [10], which are connected with striatal and limbic structures, has been implicated in MDD. Abnormalities in these areas can impair many executive functions including attention, planning, and performance monitoring [10]. Dorsal ACC-containing circuits are thought to be involved in self-evaluation, while the DLPFC is involved in processes like behavioral adjustment and attention [10]. Neurophysiological and/or anatomical changes in these regions may underlie the cognitive deficits evident in MDD.

Brain imaging studies have observed differences between depressed and healthy comparison participants in these regions. Magnetic resonance spectroscopy (MRS) studies of the DLPFC have shown altered choline concentrations in elderly MDD patients [11,12], as well as in pediatric depressed patients [13]. Mayberg et al. [14] measured glucose metabolism in depressed adults at rest, and found increased glucose metabolism in the right DLPFC [14]. In a functional magnetic resonance imaging (fMRI) study by Grimm and colleagues [15], a decrease in the blood oxygen dependent level (BOLD) signal was observed in the left DLPFC during expected and unexpected emotional judgment of photographs, and an increase in the right DLPFC during expected emotional judgment by depressed participants. To date, few studies have investigated volumetric and cortical thickness in the DLPFC in depressed youth [16] to see if these functional changes are reflected morphologically. Abe and colleagues [17] assessed gray matter volume of the prefrontal cortex in adults with MDD and noted smaller gray matter volumes in the bilateral middle frontal gyri in these individuals [17]. Non-remitting MDD adults also displayed a reduction in gray matter volume in the DLPFC [18]. Post mortem work in MDD supports these neuroimaging findings [19,20]. Peterson et al. [21] found a reduction cortical thickness across a large expanse of the lateral right cerebral hemisphere (including the DLPFC) in people at high risk for developing MDD. In summary, data from imaging studies show alterations in DLPFC function, structure and chemistry in MDD. More recently, differences in cortical thickness of the ACC have been noted in MDD (i.e., thinner medial orbital prefrontal cortex, thicker temporal pole an caudal ACC) [22] and in people at risk for developing MDD (i.e., thinner right cerebral hemisphere in persons at high risk) [21].

Finally, development itself may play a role in the disorder and adolescence is a time of special vulnerability for developing mood disorders. Physiologically, regions involved in emotional and stress regulation undergo tremendous changes during adolescence. The prefrontal cortex, hippocampus, amygdala, hypothalamus and pituitary demonstrate marked developmental trajectories [23-27] and the DLPFC is one of the last brain regions to reach maturity [28,29]. Disruptions in the normal development of these brain regions may lead to altered, and perhaps maladaptive, stress responses and associated mood disorders. Indeed, it may be that adolescents with MDD are on a different developmental trajectory than healthy youth.

In the current study, we examined cortical thickness in adolescents with MDD and healthy adolescent controls. To date, there has only been one previous report of cortical thickness in children and adolescents with MDD [16]. Cortical thickness was examined as it is sensitive to both the columnar architecture of the cortex (i.e., it spans the cortical layers) and developmental changes in normally developing and clinical populations [30-32]. We hypothesize that the children and adolescents with MDD will have thinner cortices in the middle frontal gyrus (MFG; a core portion of the DLPFC), and ACC.

## Methods

### Subjects

A total of 52 participants were recruited for this study (See Table 1). Adolescent MDD participants were recruited from hospitals, physician offices and universities in the area. Healthy control adolescent participants were recruited through advertising. A clinical interview and investigator-rated Kiddie-SADS-Present and Lifetime Version (K-SADS) then confirmed diagnoses of MDD in the affected group and classification of the healthy control participants, based on DSM-IV criteria [33]. Subject symptomology information was gathered using the Children’s Depression Rating Scale (CDRS)[34,35] and the Beck Depression Inventory (BDI). With the exception of three patients with psychiatric comorbidities, all participants were physically healthy, with IQ in the normal range (mean +/- SD) IQs and no other intellectual disorders. One MDD participant was comorbid for oppositional defiant disorder (ODD) and two suffered from substance abuse. At the time of the MRI scan, all but four MDD participants were treatment-naive. The four participants on pharmaceutical treatment prior to testing had only started the treatment within two weeks of the MRI scan. Two participants were taking sertraline [a selective serotonin reuptake inhibitor (SSRI)], one was taking moclobemide (reversible monoamine oxidase inhibitor), and the fourth was taking dextroamphetamine (psychostimulant). Exclusion criteria for participation in this study were: a history of neurological illness, medical illness, claustrophobia, >21 year of age, or the presence of a ferrous implant or pacemaker. Six MRI scans were not used in the data analyses due to poor scan quality. Unless stated otherwise, the means and standard deviations are presented for all measures. The final sample consisted of 16 controls and 30 MDD patients (see Table 1). All participants and their legal guardian provided written informed consent prior to the start of the study in accordance with the Research Ethics Board (REB) of the IWK Children’s Hospital and in compliance with the Helsinki Declaration (http://www.wma.net/en/30publications/10policies/b3/index.html).

**Table 1:** **Demographic characteristics (Mean** ± **standard deviation)**

**Item**	**Controls**	**MDD**
Age (Years)	17.19 ± 1.87	16.89 ± 2.01
Sex	7 males	9 males
	9 females	21 females
CDRS	38.87 ± 11.701	66.53 ± 14.43
BDI	1.81 ± 1.52	18.93 ± 12.98
Duration of Illness (Months)	-	35.31 ± 21.28

CDRS – Children’s Depression Rating Scale, BDI – Beck Depression Inventory.

### MRI acquisition protocol

MRI scans were conducted with a 1.5 Tesla Siemens Magnetom Vision magnetic resonance system. A sagittal scout series was acquired to test image quality. A three-dimensional fast low angle shot (FLASH) sequence was used to acquire data from 124 1.5 mm-thick contiguous coronal slices through the entire brain (echo time = 5 ms, repetition time = 25 ms, acquisition matrix = 256 × 256 pixels, field of view = 24 cm and flip angle = 40°).

### Cortical thickness analysis

Each MRI dataset underwent cortical surface reconstruction via the FreeSurfer automated cortical measurement technique. FreeSurfer is a free software program that can be downloaded (http://surfer.nmr.mgh.harvard.edu). Procedures for using the FreeSurfer program have been outlined in various sources [36-38]. First, the cortical surface undergoes reconstruction involving the correction of intensity variations that exist between MRI data sets due to variations in the magnetic field during the scanning procedure [36]. This reconstruction also involves the removal of any voxels containing the skull or other extra-cerebral entities [36]. After the intensity corrections and skull stripping, a researcher blind to the condition of each subject manually corrected the images. Subsequently, the individual data sets underwent a segmentation procedure based on a structural estimation of the gray-white matter interface [36]. In order to generate a smooth and accurate representation of both the pial surface and the gray-white interface, a triangular tessellation cover was applied to each scan, which was then inflated [36]. Inflation allows for easier visualization by reducing any interference by folding so that the cortical area within sulci can be visualized [38]. Scans were then aligned with the reference brain template based on the positioning of the gyri and sulci [38]. It is generally difficult to establish a coordinate system in which the cortical surfaces of different volumes can be accurately matched to provide consistent measurements [38]. Therefore, the reconstructed cortical surfaces were transformed into parameterizable surfaces. Parameterization provides a reproducible and consistent coordinate system [38]. Finally, the average cortical thickness measurements were taken at every point on the smoothed and aligned images [37]. Cortical thickness is calculated by averaging the distance between the pial surface and the gray-white interface [37]. It is important to note that this technique for taking automated cortical thickness measurements is not restricted by the resolution of the voxels in the original MRI data and can provide measurements accurate to a sub-millimeter scale [37]. For this study we restricted our focus to MFG and caudal ACC.

### Statistical analysis

Statistical analyses were conducted with the Statistical Package for Social Sciences (SPSS) software. The Levine’s test of variance was violated when analyzing the variance between the two study groups for the MFG (left and right: p < 0.001). Therefore, a non-parametric Mann-Whitney U test was used to analyze planned comparisons (aspects of the MFG and caudal ACC) of cortical thickness between the groups. In order to account for multiple comparisons, *p* was set at 0.01 [39]. Spearman correlations, exploratory in nature, were performed between cortical thickness and age, symptom severity, duration of illness and age of onset (for MDD patients).

## Results

### Main findings

**Figure 1:**
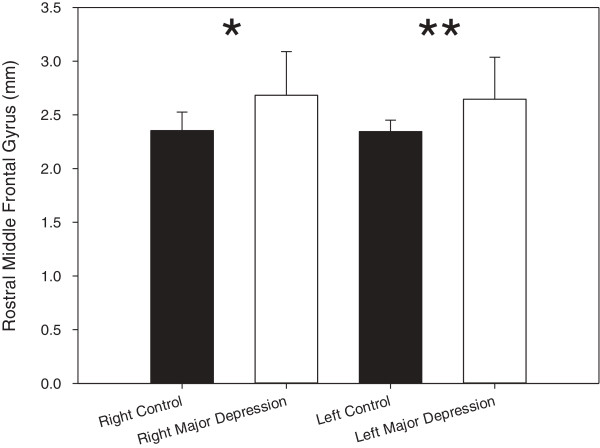
**Mean cortical thickness of the right and left rostral middle frontal gyrus (mm).** Error bars indicate standard deviation. Stars indicate significance (*p = 0.004, **p = 0.005).

**Figure 2:**
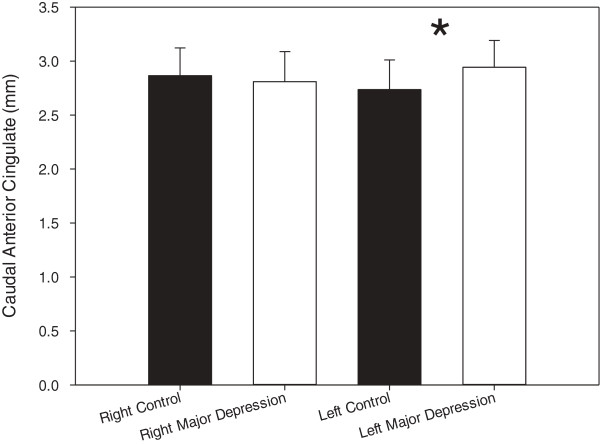
**Mean cortical thickness of the right and left caudal anterior cingulate (mm).** Error bars indicate standard deviation. Stars indicate significance (*p = 0.009).

There was no age difference between the two groups (t = 1.32, df = 51, p = 0.19). Mann-Whitney U-tests revealed the right and left MFG were thicker in MDD participants than in controls (p = 0.004 and p = 0.005 respectively; Right - controls: 2.35 ± 0.17, MDD = 2.68 ± 0.41; Left – controls: 2.34 ± 0.11, MDD: 2.65 ± 0.39), contrary to our hypothesis (Figure 1). Furthermore, the left, but not right, caudal ACC was thicker in MDD participants than in controls (p = 0.009; Left - controls: 2.74 ± 0.28, MDD: 2.94 ± 0.25; Figure 2; Right - controls: 2.77 ± 0.26, MDD: 2.80 ± 0.28). When medicated MDD participants were removed (N = 4), the results persisted (right rostral MFG: p = 0.002; left rostral MFG: p = 0.005; left caudal ACC: p = 0.008). Dropping the MDD participants with comorbidity did not significantly alter the results (right rostral MFG: p = 0.001; left rostral MFG: p = 0.001; left caudal ACC: p = 0.012). Given the unexpected nature of the results, we examined the volume output provided by FreeSurfer. Volume for the right and left MFG and left caudal ACC did not differ between groups, not did volume and thickness correlate between these regions as well. It should also be noted that removal of the two subjects with a history of substance abuse did not change the results.

### Correlations

In MDD subjects there was an inverse correlation between age and rostral MFG cortical thickness (trend for the right: r = -0.34, p = 0.07; significant for the left: r = -0.45, p = 0.01). No further correlations were found to be significant (cortical thickness and clinical variables like symptom severity, duration of illness, etc.). No significant correlations were noted in controls.

### Sex differences

In an exploratory analysis, considering the small sample size, we examined the effect of gender on the results. In the right and left rostral MFG the effect of group on cortical thickness held for females (p = 0.002 and p = 0.02, respectively) but not males. However, in the caudal ACC, the group effect noted in the left side was only seen in males (p = 0.02). Within each group, there was no difference between males and females in rostral MFC and caudal ACC thickness.

### Volume and surface area differences

In an exploratory analysis, we also examined surface area and volume. Rostral MFC and caudal ACC did not differ between groups on these measures.

## Discussion

Counter to our hypothesis, our results indicate greater cortical thickness in the MFG in children and adolescents with MDD compared with healthy controls. Additionally, cortical thickness was greater in the MDD group in the caudal ACC – similar to an effect seen in data reported by van Eijndhoven et al. [22,40] and Peterson et al. [21]. These group effects appeared to display some sex-specific effects. The implications of these results are discussed below.

The rostral middle frontal gyrus (MFG) is partially located in the dorsolateral prefrontal cortex (DLPFC) [41]. Brodmann’s area (BA) 46 makes up most of the rostral MFG, but it includes portions of BA 9 and BA 10, which comprise the DLPFC [41]. The rostral MFG is reciprocally connected to many brain regions [42]. Direct inputs and outputs include: the cingulate cortex, thalamus, orbitomedial prefrontal cortex, and posterior association cortex. Efferents connect to the hippocampus, but the DLPFC does not receive any direct input from this area. Indirect inputs to the lateral prefrontal cortex include the amygdala via the hippocampus, thalamus, cingulate cortex and the orbitomedial prefrontal cortex [42,43].

The connections and interactions of the DLPFC with other brain structures are consistent with its involvement in various complex executive functions. For instance, BA 46 and BA 9, and the lateral prefrontal cortex in general, have been implicated in working memory [44], a process often impaired in MDD [45-47]. As previously noted, alterations in prefrontal areas can also impair many executive functions including attention, planning, monitoring of performance, mood regulation and behavioral adjustment; all of can be disrupted in MDD [10,43].

As previously noted, many studies have indicated alterations in the DLPFC in MDD; from MRS studies [11-13,48], to functional imaging [14,15] and structural and cytoarchitectural research [17,49,50]. Abe and colleagues [17] found a decrease in the bilateral MFG volume in adult MDD patients. Their findings combined with our results, suggests that the MFG may be a locus of neuronal change in the context of MDD. However, it is important to note that cortical volume does not correlate with cortical thickness; a cortex with a smaller volume could indeed have a larger cortical thickness. Our finding of increased cortical thickness represents a novel finding in individuals affected by MDD. Nevertheless, post-mortem investigations of neuronal and glial size and density support our results given the inverse correlation noted between thickness and age in DLPFC in MDD participants. Both Rajkowska and colleagues [50] and Cotter and colleagues [49] found reduced size and density of both neurons and glia in the DLPFC in adults with MDD. These changes in cytoarchitecture and DLPFC volume could result from truncated pruning and/or reductions in pre-programmed cell loss during development. Pruning is essential in brain development as it limits lateral inhibition and eliminates redundant/un-needed connections. Our finding of an increase in cortical thickness that is negatively correlated with age may indicate an altered developmental trajectory than in non-depressed controls, which may exhibit characteristic pruning during development resulting in lower volumes, thinner cortices, and decreased function in the DLPFC. In people at risk for developing MDD, a thinner DLPFC was also noted [21]. The sample for that study was weighted towards adults (18% were children) and no interaction with age was detected. Cortical thickness of the DLPFC is influenced by the age of onset [51] and this may be responsible for the discrepancy noted here. Further longitudinal studies are warranted, especially in high-risk samples that develop clinical MDD.

The ACC resides in the medial frontal cortex [52] and encompasses BA 25, BA 24 and BA 32 [53]. The ACC has connections with various brain areas including the limbic system, which is critically implicated in affect [54]. Abnormal functioning of the ACC is thought to occur in anhedonia, a characteristic feature of MDD [54,55]. Neuroimaging studies have shown that the ACC is also involved in many overlapping processes as the DLPFC including working memory, language, attention and information processing [54]. The role of the ACC in conflict monitoring is believed to have a role in motivational and emotional responses, which are also altered in MDD [55]. It has been hypothesized that there is hyper-responsivity in conflict monitoring centers in MDD [55]. In depressed individuals, there is heightened ACC activity during reward anticipation [52,55]. In a study performed by Knutson et al. [56], the dorsal ACC displayed hyper-activation in depressed individuals during anticipation of a monetary reward. Our results support the finding that the ACC is over-active in youth with MDD. The caudal ACC was found to be thicker in MDD subjects, which suggests an increase in cell number and/or density. This increase could play a role in the hyperactivity evident in MDD.

Various studies have indicated that extensive neural pruning takes place in healthy adolescents [57-59]. The thicker cortex that was found in the rostral MFG and caudal ACC could reflect deficient neural pruning in depressed adolescents. MDD may be more likely in individuals with DLPFC and ACC underdevelopment (i.e., inefficient pruning) during adolescence. On their face, our finding of thicker cortex in the MFG and left caudal ACC seem to contradict findings observed in adults. The relationship with age in MDD adolescents may explain this difference. However, no correlations between cortical thickness and illness duration, age of onset, or symptom severity in adolescents with MDD emerged in the present study. Though this could indeed reflect the absence of a relationship between these variables, it is more feasible that these null effects were influenced by the small sample size and the cross-sectional nature of the study.

In the present study we found that reductions in cortical thickness of right and left rostral MFG were evident in females with MDD, but not in affected males. In the left caudal ACC, affected males were found to display reduced thickness not seen in affected females. This suggests differential regional neurodevelopment in males and females, an indication of divergent neurobiological substrates MDD in the two sexes. However, our sample size is relatively limited, thus, it is important that this finding is replicated.

The FreeSurfer program used in the current study is a reliable method for measuring cortical thickness [37]. However, FreeSurfer has not been validated for use in a pediatric population. As a result, the measurements attained in this study may not be representative of the actual cortical thicknesses in these subjects. Additionally, the four medicated participants could have influenced our cortical thickness outcomes. However, given that medication was used for a maximum of two weeks prior to the MRI scan, substantial medication-induced changes in cortical thickness were unlikely in this short time frame. Another limitation of the study is the sample size. Replication with a larger, more diverse sample is warranted. Finally, our study is limited by using a cross-sectional paradigm. Longitudinal analyses of cortical thickness and other neuroanatomical and functional parameters should be conducted into adulthood to gain a complete understanding of the implications of MDD as a developmental disorder.

## Conclusions

Due to the debilitating nature of MDD, it is imperative that the neurobiological underpinnings of this disorder be understood. To our knowledge, ours is the second study investigating cortical thickness in adolescent MDD patients. Previously, no significant differences in DLPFC or ACC cortical thicknesses were noted between MDD and control participants [16]. However, in the study by Fallucca and colleagues, the majority of the MDD participants had comorbid disorders including attention deficit hyperactivity disorder (ADHD), ODD and anxiety disorders [16], which could account for the differences in results. These discrepancies also highlight the need for further studies on cortical thickness in pediatric/adolescent MDD.

Future studies should include a larger sample size of both depressed and control subjects. It is important that MDD participants be treatment naive, and have limited comorbidity. To further validate the measurements obtained with the FreeSurfer program, it is important that this technique and others be validated for use on the pediatric population. Most importantly, in order to gain a true understanding of the developmental mechanism of MDD, it is imperative that longitudinal analyses of MDD patients be conducted throughout development.

## Competing interests

The authors have no competing interests.

## Authors’ contributions

All authors have made substantial contributions to conception and design (FPM, NC, SR), or acquisition of data (FPM, NC), or analysis and interpretation of data (SR, FPM, NJ, LML, XRY, NC); have been involved in drafting the manuscript (SR, FPM, NJ, LML, XRY, NC) or revising it critically for important intellectual content (SR, FPM, NJ, LML, XRY, NC); and have given final approval of the version to be published (SR, FPM, NJ, LML, XRY, NC). All authors read and approved the final manuscript.

## Pre-publication history

The pre-publication history for this paper can be accessed here:

http://www.biomedcentral.com/1471-244X/14/83/prepub
